# Benthic biota of Chilean fjords and channels in 25 years of cruises of the National Oceanographic Committee

**DOI:** 10.1038/s41597-023-01990-x

**Published:** 2023-02-09

**Authors:** Cristian Aldea, Leslie Novoa, María Paz Acuña, Ignacia Acevedo-Romo, Francisco Bravo

**Affiliations:** 1 Departamento de Ciencias y Recursos Naturales, Universidad de Magallanes, Punta Arenas, Chile; 2 Centro de Investigación Gaia-Antártica, Universidad de Magallanes, Punta Arenas, Chile; 3 CSIRO Chile Research Foundation, Santiago, Chile

**Keywords:** Biodiversity, Marine biology, Biogeography, Databases

## Abstract

The CIMAR program (Marine Research Cruises to Remote Areas) run by the Chilean Navy through the National Oceanographic Committee has been developed since 1995, focused on Chilean fjord and channel zones (~41–56°S; “CIMAR-Fjords”) and Chilean remote islands (“CIMAR-Islands”). Samples and data was collected on biotic and abiotic variables on all these cruises, both from the water column and benthos. Our work standardizes, compiles, and summarizes the published information on benthic organisms for twenty-one CIMAR-Fjords cruises developed in the first 25 years of the program, plus the Southern Ice Fields Cruise 1995 (precursor of the CIMAR program), which includes the distribution, abundance and geographic location of cruises sampling stations. The data set includes 8,854 records from 880 different localities, corresponding to 1,225 species from 24 different phyla (four kingdoms) and more than 150,000 individuals. Only two cruises did not record any benthic sampling. The fjords and channels of Chilean Patagonia have high biodiversity, so we hope that our data set will serve as a baseline for ecological studies and ecosystem conservation.

## Background & Summary

The Marine Research Cruises in Remote Areas (CIMAR, acronym in Spanish) have been carried out continuously since 1995, covering Chilean coastal and oceanic areas that are difficult to access and far away from the main ports of the country^1^. This is a research initiative by the Chilean Navy, developed through its National Oceanographic Committee (CONA, by its acronym in Spanish). CONA is an organization whose main function is to coordinate the institutions that carry out research and activities related to marine sciences in Chile. It currently has 31 member institutions—universities and various public and private organizations that develop marine sciences in Chile. This program has a specific research component in the Channels and Fjords of Patagonia in southern Chile (~41°S to ~56°S), called “CIMAR-Fjords Cruises”. These places require complex logistics to carry out research and are of great socioeconomic importance; until the mid-1990s there was little scientific information about them, except for some foreign explorations with little or no national participation^2–7^. An exploratory marine scientific research cruise was carried out in 1995 in marine waters adjacent to the Ice Fields^8^ coordinated by the National Oceanographic Committee, to promote basic oceanographic studies.

Numerous expeditions, the majority led by foreigners, have documented benthic species in the Patagonian zone of the fjords and channels in southern Chile, including Mollusca^9–15^, Crustacea^16–18^, Polychaeta^19,20^, and Foraminifera^21–23^ among others. Some studies have sought to advance our understanding of specific groups by offering reviews of specific taxa, biogeographical studies, catalogues, or lists of species^24–27^.

Over the course of the CIMAR program’s first 25 years, 362 research projects have been completed on its cruises, resulting in more than 400 publications in scholarly journals^1^, plus the publication of their data in annual cruise reports. The majority of this data was gathered during the so-called CIMAR-Fjords Voyages, which made up 21 of the 25 cruises of the CIMAR program, that focus in the fjords and channels of southern Chile (between 41°S and 56°S).Each research cruise included a significant amount of work on the benthic compartment, where a wide variety of benthic organisms were collected and described in both the cruise reports^8,28–46^ and several peer-review publications (e.g.^25,47–52^).

The objective of this study was to compile all the benthic records of the CIMAR-Fjords cruises, as well as the 1996 exploratory cruise to the Southern Patagonian Ice Fields. The assembled database is anticipated to serve as a baseline for new research projects and initiatives in the area. This data descriptor presents a database with 8,854 records from 880 different localities, corresponding to 1,225 species from 24 different phyla (four kingdoms) and over 150,000 individuals.

## Methods

### Study area

**Fig. 1:**
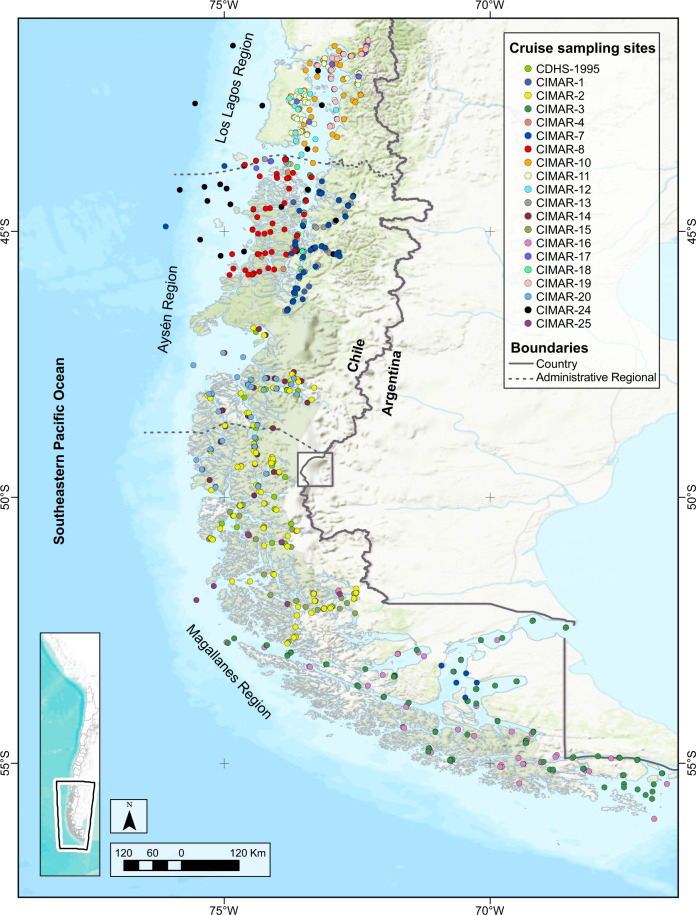
Fjords and channels in southern Chile where the CIMAR-Fjords cruises and the 1995 Southern Ice Fields Cruise (CDHS) have taken place. The study sites of each cruise are differentiated with different colours.

With a long coastline and a variety of intertidal and subtidal environments, the fjords and channels of southern Chile exhibit distinctive oceanographic conditions that are mostly explained by the fluctuating influence of oceanic, glacial, and pluvial waters. These features makes these environments highly sensitive to environmental pressures (climate change, marine pollution and fishing extraction, among other stressors)^53^. This area encompasses the Chiloense Marine and Channels and Fjords Southern Chile Ecoregions in the Magellanic Biogeographic Province^54^. A significant portion of the various environments seen in the fjords and channels of southern Chile have been described in reports and publications related to the CIMAR-Fjords Cruises (Fig. 1), including biotic and abiotic aspects, demonstrating differences in diversity and abundance of various taxa. This work covers a total of 880 locations with benthic information of biota recorded in the CIMAR-Fjords Cruises, plus the Southern Ice Fields Cruise 1995 (Fig. 1). The samples cover 25-years, from August, 1995 to October, 2019 (Table 1). Five cruises covered more than half of the total sampled sites: CIMAR-2 (114 sites), CIMAR-11 (96), CIMAR-16 (91), CIMAR-3 (82) and CIMAR-7 (77). The sites cover the three southernmost administrative regions of Chile (Los Lagos, Aysén and Magallanes), and their nine provinces. The provinces with the largest number of sites were Aysén (in Aysén Region, 221 sites), Última Esperanza Province (in Magallanes Region, 178 sites) and Chiloé (in Los Lagos Region, 101 sites), accumulating 500 sites among the three provinces.

**Table 1:** Benthic sampling statistics by research cruise from oldest to newest, and by province and region from north to south.

Cruise	Dates (y/m)	Latitudinal range	Longitudinal range	Sites
CDHS-1995	1995/08 to 1995/09	−45.06 to −50.87	−73.61 to −75.04	2 (AP-AR); 6 (CP-AR); 15 (UE-MR)
CIMAR-1	1995/11 to 1996/06	−44.29 to −46.14	−73.19 to −73.70	4 (AP-AR)
CIMAR-2	1996/10 to 1996/11	−45.21 to −52.75	−72.51 to −75.42	3 (AP-AR); 27 (CP-AR); 84 (UE-MR)
CIMAR-3	1997/10	−52.32 to −55.67	−66.77 to −74.93	2 (UE-MR); 28 (MP-MR); 24 (TF-MR); 28 (AC-MR)
CIMAR-4	1999/02 to 1999/03	−43.90 to −46.50	−73.10 to −74.30	8 (AP-AR)
CIMAR-7	2001/07	−43.65 to −53.77	−70.25 to −76.10	2 (CH-LR); 70 (AP-AR); 3 (MP-MR)*; 2 (TF-MR)*
CIMAR-8	2002/07 to 2002/09	−43.65 to −45.83	−73.35 to −74.90	2 (CH-LR); 44 (AP-AR)
CIMAR-9	WBS			
CIMAR-10	2004/08 to 2004/11	−41.51 to −43.74	−72.33 to −74.13	15 (LP-LR); 25 (CH-LR); 17 (PP-LR); 5 (AP-AR)
CIMAR-11	2005/07 to 2005/11	−41.41 to −43.87	−72.30 to −74.12	21 (LP-LR); 53 (CH-LR); 20 (PP-LR); 2 (AP-AR)
CIMAR-12	2006/07 to 2006/11	−41.55 to −44.66	−72.33 to −73.80	8 (LP-LR); 9 (CH-LR); 5 (PP-LR); 2 (AP-AR)
CIMAR-13	2007/07 to 2007/11	−43.65 to −46.20	−72.61 to −74.63	1 (CH-LR); 41 (AP-AR)
CIMAR-14	2008/10 to 2008/11	−46.84 to −50.16	−73.43 to −75.41	2 (AP-AR); 21 (CP-AR); 18 (UE-MR)
CIMAR-15	2009/10 to 2009/11	−50.11 to −52.75	−72.53 to −75.28	39 (UE-MR)
CIMAR-16	2010/10 to 2010/11	−51.75 to −56.04	−66.68 to −74.96	1 (UE-MR); 25 (MP-MR); 19 (TF-MR); 46 (AC-MR)
CIMAR-17	2011/10	−41.42 to −43.82	−72.29 to −74.40	12 (LP-LR); 2 (CH-LR); 4 (PP-LR); 2 (AP-AR)
CIMAR-18	2012/06 to 2012/07	−43.79 to −46.48	−72.83 to −73.80	17 (AP-AR)
CIMAR-19	2013/07	−41.42 to −43.03	−72.29 to −73.27	17 (LP-LR); 2 (CH-LR); 6 (PP-LR)
CIMAR-20	2014/10	−47.29 to −49.81	−73.63 to −75.57	24 (CP-AR); 13 (UE-MR)
CIMAR-23	WBS			
CIMAR-24	2018/09 to 2018/10	−41.51 to −45.47	−72.57 to −75.83	2 (LP-LR); 5 (CH-LR); 19 (AP-AR)
CIMAR-25	2019/09 to 2019/10	−50.71 to −52.01	−72.83 to −75.52	6 (UE-MR)

Key: Los Lagos Region (LR), Aysén Region (AR), Magallanes Region (MR), Llanquihue Province (LP), Chiloé Province (CH), Palena Province (PP), Aysén Province (AP), Capitán Prat Province (CP), Última Esperanza Province (UE), Magallanes Province (MP), Tierra del Fuego Province (TF), Antártica Chilena Province (AC); WBS (Without benthic samples reported). More information about the cruises in the book of 25 years of CIMAR cruises^1^.

*Sampling sites in another locality different from the study area of the cruise.

### Types of sampling and preservation

The database contains records of 8854 occurrences of benthic species. Samples were collected using the following methods and sampling gears: Agassiz or modified Agassiz trawl (3625 occurrences), Box corer (3010), Scuba and intertidal sampling (1097), Van Veen and other combined sampling devices (874) and undetermined sampling gears (248).

**Fig. 2:**
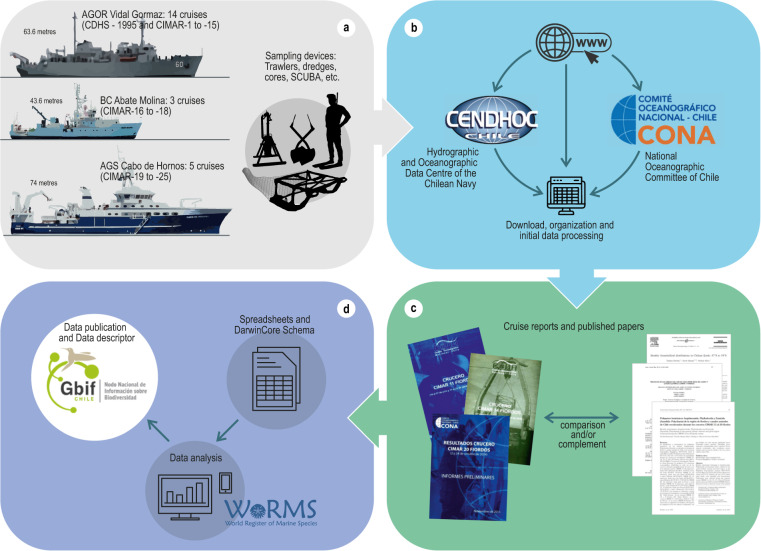
Stages of information processing from raw cruise data: (**a**), information sources and initial treatment; (**b**), data extraction from cruise reports and scientific papers; (**c**) DarwinCore standardization, analysis and publication of the database (**d**).

The record book of the first 25 years of the CIMAR cruises^1^ was used to identify the many studies that reported benthic biota observations, from the intertidal to the deep sea (Fig. 2a). The reports of the 1995 Southern Ice Fields Cruise^8^ were also investigated to identify benthic dataset. The data were selected from official information sources (Hydrographic and Oceanographic Data Centre of the Chilean Navy and National Oceanographic Committee of Chile) by searching for articles on the World Wide Web; all information sources were downloaded, organized and systematized (Fig. 2b). In the case of the World Wide Web, the Google Scholar and Web of Science portals were used and the search strategy consisted of systematically using keyword combinations (e.g. “CIMAR-Fjords”, “CIMAR-Fiordos”, benthic, benthos, [main taxa] and any other derivations and combinations of terms that may be necessary). For each corresponding paper or report, all the records of the declared benthic biota were extracted, comparing and/or complementing the records present in both reports and papers (Fig. 2c). All occurrence records were tabulated and arranged in spreadsheets according to the DarwinCore standard^55,56^. Then, the entire data set was analysed, and the taxonomy updated as required in accordance with the World Register of Marine Species^57^. Finally, the dataset was published in GBIF through the Integrated Publishing Toolkit (Fig. 2d).

## Data Records

The data were recorded under the DarwinCore standard^55,56^ in a matrix named “Benthic biota of CIMAR-Fiordos and Southern Ice Field Cruises”^58^. The occurrence dataset contains direct basic information (description, scope [temporal, geographic and taxonomic], methodology, bibliography, contacts, data description, GBIF registration and citation), project details, metrics (taxonomy and occurrences classification), activity (citations and download events) and download options. The following data fields were occupied:

Column 1: “occurrenceID” (single indicator of the biological record indicating the cruise and correlative record).

Column 2: “basisOfRecord” (“PreservedSpecimen” for occurrence records with catalogue number of scientific collection, “MaterialCitation” for any literature record).

Column 3: “institutionCode” (The acronym in use by the institution having custody of the sample or information referred to in the record).

Column 4: “collectionCode” (The name of the cruise).

Column 5: “catalogNumber” (The repository number in museums or correlative number).

Column 6: “type” (All records entered as “text”).

Column 7: “language” (Spanish, English or both).

Column 8: “institutionID” (The identifier for the institution having custody of the sample or information referred to in the record).

Column 9: “collectionID” (The identifier for the collection or dataset from which the record was derived).

Column 10: “datasetID” (The code “CONA-benthic-biota-database” for entire database).

Column 11: “recordedBy” (Author/s who recorded the original occurrence [publication source]).

Column 12: “individualCount” (Number of individuals recorded).

Column 13: “associatedReferences” (Publication source [report and/or paper/s] for each record).

Column 14: “samplingProtocol” (The sampling gear for each record).

Column 15: “eventDate” (The date-time or interval during which the record occurred).

Column 16: “eventRemarks” (Comments or notes about the event).

Column 17: “continent” (Location).

Column 18: “country” (Location).

Column 19: “countryCode” (The standard code for the country in which the location occurs).

Column 20: “stateProvince” (Location, refers to the Administrative Region of Chile).

Column 21: “county” (Location, refers to the Administrative Province of Chile).

Column 22: “municipality” (Location, refers to the Administrative Commune of Chile).

Column 23: “locality” (The specific name of the place).

Column 24: “verbatimLocality” (The original textual description of the place).

Column 25: “verbatimDepth” (The original description of the depth).

Column 26: “minimumDepthInMeters” (The shallowest depth of a range of depths).

Column 27: “maximumDepthInMeters” (The deepest depth of a range of depths).

Column 28: “locationRemarks” (The name of the sample station of the cruise).

Column 29: “verbatimLatitude” (The verbatim original latitude of the location).

Column 30: “verbatimLongitude” (The verbatim original longitude of the location).

Column 31: “verbatimCoordinateSystem” (The coordinate format for the “verbatimLatitude” and “verbatimLongitude” or the “verbatimCoordinates” of the location).

Column 32: “verbatimSRS” (The spatial reference system [SRS] upon which coordinates given in “verbatimLatitude” and “verbatimLongitude” are based)

Column 33: “decimalLatitude” (The geographic latitude in decimal degrees).

Column 34: “decimalLongitude” (The geographic longitude in decimal degrees).

Column 35: “geodeticDatum” (The spatial reference system [SRS] upon which the geographic coordinates given in “decimalLatitude” and “decimalLongitude” was based).

Column 36: “coordinateUncertaintyInMeters” (The horizontal distance from the given “decimalLatitude” and “decimalLongitude” describing the smallest circle containing the whole of the location).

Column 37: “georeferenceRemarks” (Notes about the spatial description determination).

Column 38: “identifiedBy” (Responsible for recording the original occurrence [publication source]).

Column 39: “dateIdentified” (The date-time or interval during which the identification occurred.)

Column 40: “identificationQualifier” (A taxonomic determination [e.g., “sp.”, “cf.”]).

Column 41: “scientificNameID” (An identifier for the nomenclatural details of a scientific name).

Column 42: “scientificName” (The name of species or taxon of the occurrence record).

Column 43: “kingdom” (The scientific name of the kingdom in which the taxon is classified).

Column 44: “phylum” (The scientific name of the phylum or division in which the taxon is classified).

Column 45: “class” (The scientific name of the class in which the taxon is classified).

Column 46: “order” (The scientific name of the order in which the taxon is classified).

Column 47: “family” (The scientific name of the family in which the taxon is classified).

Column 48: “genus” (The scientific name of the genus in which the taxon is classified).

Column 49: “subgenus” (The scientific name of the subgenus in which the taxon is classified).

Column 50: “specificEpithet” (The name of the first or species epithet of the “scientificName”).

Column 51: “infraspecificEpithet” (The name of the lowest or terminal infraspecific epithet of the “scientificName”).

Column 52: “taxonRank” (The taxonomic rank of the most specific name in the “scientificName”).

Column 53: “scientificNameAuthorship” (The authorship information for the “scientificName” formatted according to the conventions of the applicable nomenclatural Code).

Column 54: “verbatimIdentification” (A string representing the taxonomic identification as it appeared in the original record).

The information sources (see Fig. 2b) provided a total of 107 publications (22 cruise reports and 85 scientific papers; see Fig. 2c). Nineteen of the 22 cruise reports reviewed provided species occurrence records^8,28–30,32–46^, one provided qualitative or descriptive data, with no recorded occurrences^31^, and two did not provide information on benthic biota (CIMAR-9 and −23 cruises). Of all the scientific papers reviewed, 74 provided records of species occurrences (Table 2), while 11 did not provide any record, as they were data without occurrences of geographically referenced species or with descriptive or qualitative information: Foraminifera^59–62^, Annelida^63–66^, Fishes^67^, Mollusca^68^ and Echinodermata^69^. The phyla with the highest number of publications were the following: Annelida (present in 18 reports and 21 papers), Mollusca (in 14 and 20), Arthropoda (in 10 and 18), Echinodermata (in 10 and 9), Chordata (in 10 and 9) and Foraminifera (in 4 and 10).

**Table 2:** Publications with >100 occurrences, indicating the main recorded taxa.

“RecordedBy” in database	Main taxa	Occurrences
Hromic *et al*. (2006)^25^ **	Foraminifera	988
Thatje & Brown (2009)^48^ **	Annelida, Arthropoda, Mollusca, others	431
Arellano *et al*. (2011)^49^ ***	Foraminifera	407
Rodríguez-Villegas *et al*. (2021)^52^ *	Myzozoa	375
Rozbaczylo *et al*. (2017)^51^ *	Annelida	357
Seguel *et al*. (2015)^50^	Myzozoa	297
Mutschke (2006)^70^ ***	Echinodermata, Annelida, Mollusca, others	288
Ríos *et al*. (2005)^71^ ***	Echinodermata, Annelida, Mollusca, others	286
Zapata-Hernández *et al*. (2016)^72^ *	Echinodermata, Annelida, Mollusca, others	227
Valdovinos *et al*. (2008)^73^ ***	Mollusca	220
Cárdenas *et al*. (2008)^47^ ***	Mollusca	197
Mansilla *et al*. (2013)^74^ **	Ochrophyta, Rhodophyta, Chlorophyta	195
Mutschke *et al*. (2017)^75^	Echinodermata	179
Osorio *et al*. (2006)^76^ ***	Mollusca	150
Ríos *et al*. (2013)^77^ *	Echinodermata, Mollusca, Arthropoda, others	138
Soto *et al*. (2012)^78^ ***	Rhodophyta, Ochrophyta, Mollusca, others	118
Osorio & Reid (2004)^79^ ***	Mollusca	108
Hromic (2011a)^80^ **	Foraminifera	103
Retamal (2007a)^81^ ***	Arthropoda	103
Montiel San Martín (2005)^24^ **	Annelida	101

*Occurrences also partially recorded in corresponding cruise reports^8,28–30,32–46^.

**Occurrences also partially recorded in other publications.

***Occurrences also partially recorded in corresponding cruise reports^8,28–30,32–46^ and other publications.

**Fig. 3:**
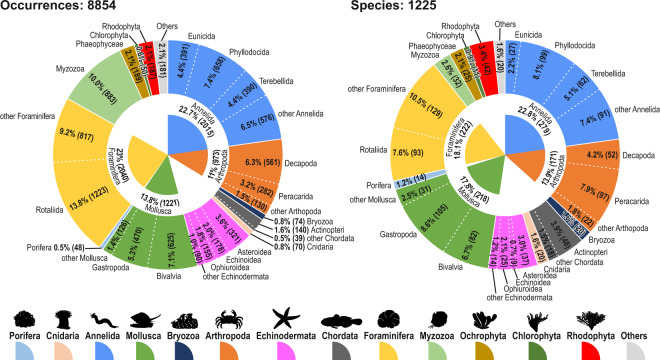
Occurrences and total species by taxon, considering large taxonomic groups of the benthic biota recorded in the CIMAR 1 to 25 and CDHS-1995 cruises. The absolute values of occurrences and species are represented in parentheses.

The information registry includes data on occurrences and number of individuals for 8,854 records (files in the database), representing 1,225 species (Fig. 3). The main taxa in terms of occurrence and number of species were Annelida (mainly Polychaeta), Foraminifera, Mollusca and Arthopoda (mainly Crustacea), together accumulating ~70% of total occurrences and ~73% of the total species (Fig. 3). The large number of recorded occurrences of Myzozoa (10%) should be highlighted, which, however, only represent about 32 species. Echinodermata represented ~8% of occurrences and 7% of species.

**Fig. 4:**
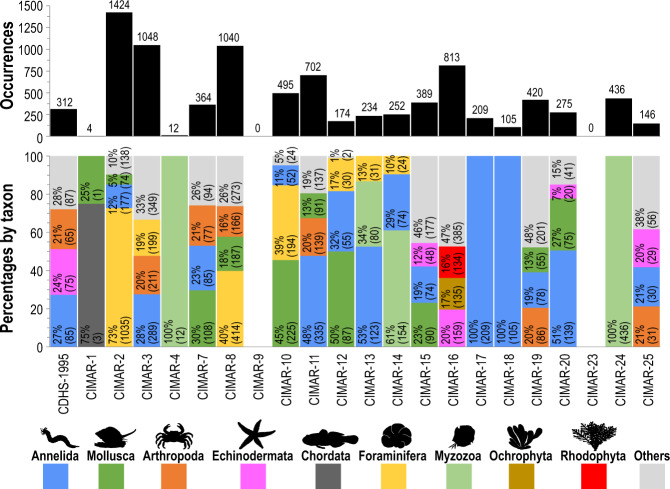
Total occurrences and percentages per dominant taxon recorded in each of the CIMAR 1 to 25 and CDHS-1995 cruises. The absolute values of occurrences per dominant taxon are represented in parentheses.

**Fig. 5:**
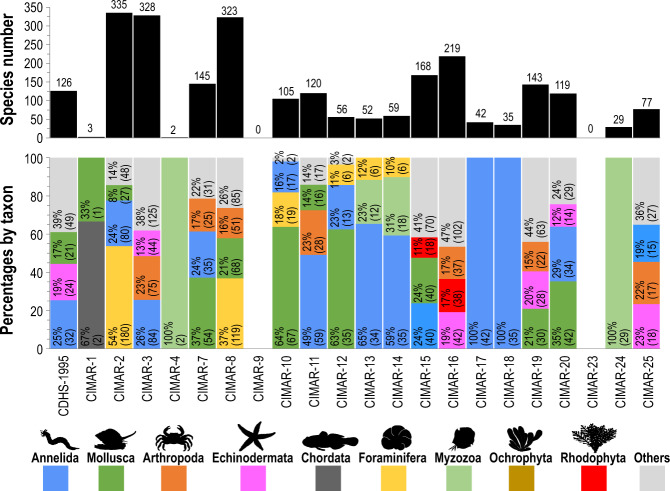
Total species and percentages per dominant taxon recorded in each of the CIMAR 1 to 25 and CDHS-1995 cruises. The absolute values of species per dominant taxon are represented in parentheses.

The cruises with the highest number of occurrences were CIMAR-2 (with 1,424), followed by CIMAR-8 (1,040) and CIMAR-16 (813) (Fig. 4). Three dominant taxonomic groups were recorded in most cruises, except for cruises CIMAR-1, CIMAR-4, CIMAR-17, CIMAR-18 and CIMAR-24 (Fig. 4). The cruises with the highest number of species recorded were CIMAR-2 (with 335), CIMAR-3 (328) and CIMAR-8 (323) (Fig. 5). Three or fewer dominant taxonomic groups were recorded only in the CIMAR-1, CIMAR-4, CIMAR-17, CIMAR-18 and CIMAR-24 cruises (Fig. 5).

**Fig. 6:**
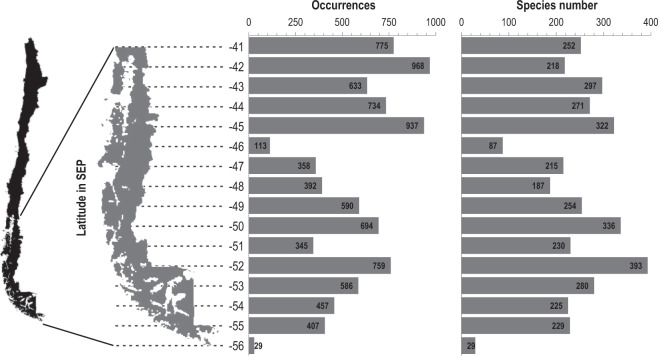
Occurrences and number of species recorded by latitudinal band from the CIMAR 1 to 25 and CDHS-1995 cruises. SEP: South-eastern Pacific.

The latitudinal bands 42°S and 45°S are those with the highest number of occurrences (Fig. 6), while the 56°S and 46°S bands had the fewest. The highest number of species was recorded in the 52°S and 50°S latitudinal bands, while, as with the occurrences, the lowest values corresponded to the 56°S and 46°S latitudinal bands (Fig. 6).

## Technical Validation

The biodiversity data was formatted according to the Darwin Core standard^55,56^ of the GBIF platform. Each occurrence was entered according to its record in all the sources of information present: cruise reports and/or publications. Then each occurrence checked against the original coordinates published in the cruise reports. The depth data, sampling gear, individual count and –when applicable– the biological collection repository number were also compared between the primary information sources (cruise reports) and publications. A considerable number of occurrences were corrected after this comparison (total: 302 occurrences), mainly by adjusting their coordinates or other location data; these modifications were noted in the database in the “georeferenceRemarks” or “eventRemarks” column, depending on the nature of the modification. A total of 77 occurrences did not record geographic location (latitude and longitude) and could not be inferred from any source. Finally, all the nomenclature was verified by using World Register of Marine Species^57^, “WoRMS Taxon match” tool.

## Data Availability

No custom code was used.

## References

[1] Comité Oceanográfico Nacional. *CIMAR 25 años. Cruceros de Investigación Marina en Áreas Remotas*. (Servicio Hidrográfico y Oceanográfico de la Armada de Chile, 2021).

[2] Fitzroy, R., King, P. P. & Darwin, C. *Narrative of the surveying voyages of His Majesty’s ships Adventure and Beagle, between the years 1826 and 1836, describing their examination of the southern shores of South America, and the Beagle’s circumnavigation of the globe*. (H. Colburn, 1839).

[3] Martial, L. F. *Histoire du voyage. Mission Scientifique du Cap Horn, 1882–1883*. **vol. 1** (Ministère de la Marine and Ministère de l’Instruction Publique, 1888).

[4] Murray, J. *A summary of the scientific results obtained at the sounding, dredging and trawling stations of HMS Challenger*. **vol. 1** (HM Stationery Office, 1885).

[5] Holdgate MW (1960). The Royal Society Expedition to southern Chile. Proc. R. Soc. Lond. B..

[6] Brattström H, Johanssen A (1983). Ecological and regional zoogeography of the marine benthic fauna of Chile. Sarsia.

[7] Arntz WE, Gorny M (1996). Cruise Report of the Joint Chilean-German-Italian Magellan ‘Victor Hensen’ Campaign in 1994. Ber Polar- Meeresforsch.

[8] Comité Oceanográfico Nacional. *Crucero de Investigación Científica Marina a los Fiordos y Canales adyacentes a Campos de Hielo Sur (18 de agosto - 4 de septiembre de 1995)*. 1–122 (Secretaría Ejecutiva del Comité Oceanográfico Nacional, 1995).

[9] King PP, Broderip WJ (1832). Description of the Cirrhipeda, Conchifera and Mollusca, in a collection formed by the officies of HMS Adventure and Beagle employed between the years 1826 and 1830 in surveying the southern coast of South America including the Straits of Magalhaens and the coast of Tierra del Fuego. Zoological Journal.

[10] Rochebrune, A. T. & Mabille, J. Mollusques. in *Mission Scientifique du Cap Horn, 1882–1883*. **vol. 6** 1–129 (Ministère de la Marine and Ministère de l’Instruction Publique, 1889).

[11] Strebel H (1904). Beiträge zur Kenntnis der Molluskenfauna der Magalhaen-Provinz. Zool. Jahrb., Abt. Syst. Geogr. Biol. Tiere.

[12] Soot-Ryen, T. *Reports of the Lund University Chile Expedition, 1948–49, 35. Pelecypoda*. Avd. 2. Bd 55. Nr 6. (Lunds Universitets Arsskrift. N.F., 1959).

[13] Leloup, E. *Reports of the Lund University Chile Expedition, 1948–49, 27. Polyplacophora*. Avd. 2. Bd 52. Nr 15. (Lunds Universitets Arsskrift. N.F., 1956).

[14] Dell RK (1971). The marine Mollusca of the Royal Society Expedition to Southern Chile, 1958–59. Rec. Dom. Mus..

[15] Linse, K. *The shelled Magellanic Mollusca: with special reference to biogeography relations in the Southern Ocean*. (A.R.G. Gantner Verlag KG, 2002).

[16] Milne-Edward, A. Crustacés. In *Mission Scientifique du Cap Horn, 1882–1883*. vol. 6 1-76. pl. 1–8 (Ministère de la Marine and Ministère de l’Instruction Publique, 1891).

[17] Haig, J. *Reports of the Lund University Chile Expedition, 1948–49, 20. The Crustacea Anomura of Chile*. Avd. 2. Bd 51. Nr 12. (Lunds Universitets Arsskrift. N.F., 1955).

[18] Garth JS, Haig J, Yaldwyn JC (1967). The Decapod Crustacea of the Royal Society Expedition to Southern Chile, 1958–59. Trans. Roy. Soc. New Zealand.

[19] Fauvel P (1941). Annélides Polychètes de la Mission du Cap Horn (1882–1883). Bull. Mus. natl. hist. nat..

[20] Wesenberg-Lund, E. *Reports of the Lund University Chile Expedition 1948–49, 43. Polychaeta Errantia*. Avd. 2. Bd 57. Nr 12. (Lunds Universitets Arsskrift. N.F., 1962).

[21] Brady, H. B. *Report on the foraminifera dredged by HMS Challenger during the years 1873–1876*. **vol. 9** (Ehrenmitglied des naturwissenschaftlichen Vereins, Liineburg; Minister of the Free church of Scotland, 1884).

[22] Ishman SE, Martinez R (1995). Distribution of modern benthic foraminifers from the fjord region of southern Chile (42 S to 55 S). Antarctic Journal of the United States.

[23] Violanti D, Loi B, Melis R (2000). & others. Distribution of Recent Foraminifera from the Strait of Magellan. First quantitative data. Boll. Mus. Regionale. Sci. Nat. Torino.

[24] Montiel San Martín A (2005). Biodiversity, zoogeography and ecology of polychaetes from the Magellan region and adjacent areas = Diversität, Zoogeographie und Ökologie von Polychaeten der Magellanregion und angrenzender Gebiete. Ber Polar- Meeresforsch.

[25] Hromic T, Ishman S, Silva N (2006). Benthic foraminiferal distributions in Chilean fjords: 47°S to 54°S. Mar. Micropaleontol..

[26] Esquete, P. & Aldea, C. Benthic Peracarids (Crustacea) from an unexplored area of Patagonian channels and Fjords. *Biodivers. Data J*. **8** (2020). 10.3897/BDJ.8.e58013 PMC7536244 33061780

[27] Häussermann, V. & Försterra, G. *Marine benthic fauna of Chilean Patagonia: illustrated identification guide*. (Nature in Focus, 2009).

[28] CONA. *Taller sobre los resultados del Crucero Cimar-Fiordo 1. Libro de Resúmenes*. (Comité Oceanográfico Nacional, 1996).

[29] CONA. *Taller sobre los resultados del Crucero Cimar-Fiordo 2. Libro de Resúmenes*. (Comité Oceanográfico Nacional, 1997).

[30] CONA. *Taller sobre los resultados del Crucero Cimar-Fiordo 3. Libro de Resúmenes*. (Comité Oceanográfico Nacional, 1999).

[31] CONA. *Taller sobre los resultados del Crucero Cimar-Fiordo 4. Libro de Resúmenes*. (Comité Oceanográfico Nacional, 1999).

[32] CONA. *Taller sobre los resultados del Crucero Cimar 7 - Fiordos. Libro de Resúmenes*. (Comité Oceanográfico Nacional, 2002).

[33] CONA. *Taller sobre los resultados del Crucero Cimar 8 - Fiordos. Libro de Resúmenes*. (Comité Oceanográfico Nacional, 2003).

[34] CONA. *Taller sobre los resultados del Crucero Cimar 10 - Fiordos. Libro de Informes Preliminares*. (Comité Oceanográfico Nacional, 2005).

[35] CONA. *Resultados Crucero Cimar 11 - Fiordos. Libro de Informes Preliminares*. (Comité Oceanográfico Nacional, 2006).

[36] CONA. *Resultados Crucero Cimar 12 - Fiordos. Libro de Informes Preliminares*. (Comité Oceanográfico Nacional, 2007).

[37] CONA. *Resultados Crucero Cimar 13 - Fiordos. Libro de Informes Preliminares*. (Comité Oceanográfico Nacional, 2008).

[38] CONA. *Resultados Crucero Cimar 14 - Fiordos. Libro de Informes Preliminares*. (Comité Oceanográfico Nacional, 2009).

[39] CONA. *Resultados Crucero Cimar 15 - Fiordos. Libro de Informes Preliminares*. (Comité Oceanográfico Nacional, 2010).

[40] CONA. *Resultados Crucero Cimar 16 - Fiordos. Libro de Informes Preliminares*. (Comité Oceanográfico Nacional, 2011).

[41] CONA. *Resultados Crucero Cimar 17 - Fiordos. Libro de Informes Preliminares*. (Comité Oceanográfico Nacional, 2012).

[42] CONA. *Resultados Crucero Cimar 18 - Fiordos. Libro de Informes Preliminares*. (Comité Oceanográfico Nacional, 2013).

[43] CONA. *Resultados Crucero Cimar 19 - Fiordos. Libro de Informes Preliminares*. (Comité Oceanográfico Nacional, 2014).

[44] CONA. *Resultados Crucero Cimar 20 - Fiordos. Libro de Informes Preliminares*. (Comité Oceanográfico Nacional, 2015).

[45] CONA. *Resultados Crucero Cimar 24 - Fiordos. Libro de Informes Preliminares*. (Comité Oceanográfico Nacional, 2019).

[46] CONA. *Resultados Crucero Cimar 25 - Fiordos. Libro de Informes Preliminares*. (Comité Oceanográfico Nacional, 2021).

[47] Cárdenas J, Aldea C, Valdovinos C (2008). Chilean marine mollusca of Northern Patagonia collected during the CIMAR-10 Fjords Cruise. Gayana.

[48] Thatje S, Brown A (2009). The macrobenthic ecology of the Straits of Magellan and the Beagle Channel. An. Inst. Patagon..

[49] Arellano F, Quezada L, Olave C (2011). Familia Cassidulinidae (Protozoa: Foraminiferida) en canales y fiordos patagónicos chilenos. An. Inst. Patagon..

[50] Seguel M, Díaz PA, Labra G (2015). Patrones de distribución y abundancia de quistes de dinoflagelados en sedimentos recientes de la Patagonia chilena. Cienc. Tecnol. Mar.

[51] Rozbaczylo N, Vásquez-Yáñez P, Moreno RA, Díaz-Díaz O (2017). Poliquetos bentónicos Amphinomida, Phyllodocida y Eunicida (Annelida: Polychaeta) de la región de fiordos y canales australes de Chile recolectados durante los cruceros CIMAR 13 al 20 fiordos. An. Inst. Patagon..

[52] Rodríguez-Villegas C (2021). Drivers of dinoflagellate benthic cyst assemblages in the NW Patagonian Fjords System and its adjacent oceanic shelf, with a focus on harmful species. Sci. Total Environ..

[53] Fernández M (2000). Diversity, dynamics and biogeography of Chilean benthic nearshore ecosystems: an overview and guidelines for conservation. Rev. Chil. de Hist. Nat..

[54] Spalding MD (2007). Marine ecoregions of the world: a bioregionalization of coastal and shelf areas. BioScience.

[55] Wieczorek J (2012). Darwin Core: An Evolving Community-Developed Biodiversity Data Standard. PLoS ONE.

[56] Plata, C., Buitrago, L., Ortiz, R., Díaz, J. & Escobar, D. Plantilla para la publicación de registros biológicos. V. 3.5. (2020).

[57] WoRMS Editorial Board. World Register of Marine Species. Available from https://www.marinespecies.org at VLIZ. Accessed 2022-06-14. 10.14284/170 (2022).

[58] Aldea C, Novoa L, Acuña MP, Bravo F (2022). GBIF.

[59] Hromic T, Zuñiga M (2003). Foraminíferos (Protozoa: Foraminifera) de la superfamilia Buliminacea Jones 1875, en canales y fiordos Patagónicos, Chile. An. Inst. Patagon..

[60] Hromic T (2006). Distribución latitudinal de foraminíferos bentónicos (Protozoa: Foraminiferida) a nivel de subórdenes y familias, en canales y fiordos patagónicos chilenos. Invest. Mar., Valparaíso.

[61] Hromic T (2011). Foraminíferos bentónicos recolectados durante la expedición CIMAR 14 FIORDOS, Patagonia Chilena. An. Inst. Patagon..

[62] Hromic T (2012). Foraminíferos bentónicos de la expedición CIMAR 11 FIORDOS, Canales Patagónicos Chilenos: Biodiversidad y abundancia. Cienc. Tecnol. Mar.

[63] Montiel A, Gerdes D, Arntz W (2005). Polychaete assemblages on the Magellan and Weddell Sea shelves: comparative ecological evaluation. Mar. Ecol. Prog. Ser..

[64] Montiel A, Gerdes D, Arntz W (2005). Distributional patterns of shallow-water polychaetes in the Magellan region: a zoogeographical and ecological synopsis. Sci. Mar..

[65] Rozbaczylo N, Moreno R, Díaz-Díaz O, Martínez S (2006). Poliquetos bentónicos submareales de fondos blandos de la región de Aysén, Chile: Clado Terebellida (Annelida, Polychaeta). Cienc. Tecnol. Mar.

[66] Rozbaczylo N, Moreno R, Sepúlveda R, Carrasco F, Mariscal J (2009). Poliquetos bentónicos de los Fiordos Magallánicos desde el Seno Reloncaví hasta el Golfo de Corcovado (Chile). Cienc. Tecnol. Mar.

[67] Pequeño G, Olivera F (2007). Peces litorales de los canales de Aysén, capturados durante los dos cruceros del proyecto CIMAR 9 FIORDOS, en el año 2003. Cienc. Tecnol. Mar.

[68] Osorio C, Reid DG, Ramajo L (2005). Moluscos en los canales del Sur de Chile entre Boca del Guafo y Estero Elefantes (Cimar 7 Fiordos). Cienc. Tecnol. Mar.

[69] Mutschke E, Rios C, Aldea C, Montiel A, Silva F (2016). Biodiversidad marina en Magallanes: equinodermos del Pabellón de Colecciones Edmundo Pisano V. An. Inst. Patagon..

[70] Mutschke, E. Biodiversidad y estructura de la comunidad macrobentónica en canales y fiordos australes. in *Avances en el conocimiento oceanográfico de las aguas interiores chilenas, Puerto Montt a cabo de Hornos* (eds. Silva, N. & Palma, S.) (Comité Oceanográfico Nacional, 2006).

[71] Ríos C, Mutschke E, Montiel A, Gerdes D, Arntz WE (2005). Soft-bottom macrobenthic faunal associations in the southern Chilean glacial fjord complex. Sci. Mar..

[72] Zapata-Hernández G (2016). Community structure and trophic ecology of megabenthic fauna from the deep basins in the Interior Sea of Chiloé, Chile (41–43° S). Cont. Shelf Res..

[73] Valdovinos C (2008). Biodiversidad marina en el norte de la Provincia Magallánica (43° 49′−41° 32′ S): composición y patrones espaciales de diversidad de moluscos submareales. Cienc. Tecnol. Mar.

[74] Mansilla A (2013). Macroalgas Marinas Bentónicas del Submareal Somero de la Ecorregión Subantártica de Magallanes, Chile. An. Inst. Patagon.

[75] Mutschke E, Gerdes D, Ríos C (2017). Distribution and abundance patterns of echinoderms in the fjord and channel complex from a subantarctic north Patagonian Ice field, Magellan region. Rev. Biol. Trop..

[76] Osorio C, Peña R, Ramajo L, Garcelón N (2006). Malacofauna bentónica de los canales oceánicos del Sur de Chile (43° −45° S). Cienc. Tecnol. Mar.

[77] Ríos C, Mutschke E, Montiel A (2013). Composición y Estructura de la Comunidad Macrobentónica en el Sitema Interior de Canales y Fiordos del Extremo Austral de Chile. An. Inst. Patagon.

[78] Soto EH (2012). Biotopos marinos intermareales entre Canal Trinidad y Canal Smyth, Sur de Chile. Rev. Biol. Mar. Oceanogr..

[79] Osorio C, Reid DG (2004). Moluscos marinos intermareales y submareales entre la Boca del Guafo y el Estero Elefantes, sur de Chile. Invest. Mar., Valparaíso.

[80] Hromic T (2011). Análisis de la comunidad foraminiferológica bentónica del seno Reloncaví, islas Desertores, golfo Ancud y golfo Corcorvado, Chile. Cienc. Tecnol. Mar.

[81] Retamal MA (2007). Nota sobre la biodiversidad carcinologica (Stomatopoda y Decapoda) en los fiordos occidentales entre la boca del Guafo y estero Elefantes. Cienc. Tecnol. Mar.

